# AI in psoriatic disease (“PsAI”): Current insights and future directions

**DOI:** 10.1007/s10067-025-07418-8

**Published:** 2025-04-02

**Authors:** Vincenzo Venerito, Fabian Proft

**Affiliations:** 1 Rheumatology Unit - Department of Precision and Regenerative Medicine and Ionian Area, University of Bari “Aldo Moro”, Bari, Italy; 2 Department of Gastroenterology, Infectiology and Rheumatology (Including Nutrition Medicine), Charité-Universitätsmedizin Berlin, Berlin, Germany

Rheumatic diseases remain among the most intricate challenges in modern medicine, their complexity reflected in elaborate pathophysiological interactions, diverse clinical manifestations, and significant heterogeneity in treatment responses. This complexity is particularly evident in psoriatic disease (PsD), where the interplay between genetic predisposition, environmental triggers, and immune dysregulation creates a diagnostic and therapeutic puzzle [1–4]. Patients may experience a wide range of skin and musculoskeletal symptoms that can mimic other conditions, while certain disease domains, such as axial involvement, remain difficult to assess. In such a context, the potential value of artificial intelligence (AI) becomes increasingly evident, not simply as a substitute for human expertise, but rather as an ally that excels in systematically untangling complexity and illuminating the latent patterns hidden within extensive, multidimensional datasets.

Predictive modeling stands at the forefront of AI’s promise in PsD management, offering the potential to anticipate outcomes and guide more informed therapeutic decision-making (Fig. 1). By applying supervised learning techniques to large patient datasets, models can be trained on known outcomes—such as the efficacy of a particular biological therapy [5] or the likelihood of a comorbidity, including cardiovascular events—and then used to forecast these outcomes in new patients based on their clinical and demographic characteristics. Early work in this area has been encouraging, with some predictive models achieving impressive internal validation accuracies of up to 85% in cardiovascular risk prediction [6]. These models derive their power from extensive inputs ranging from traditional clinical data (such as disease duration, joint counts, and treatment history) to more complex variables like imaging findings, laboratory biomarkers, and even patient-generated information from wearable sensors and mobile health applications.

**Fig. 1:**
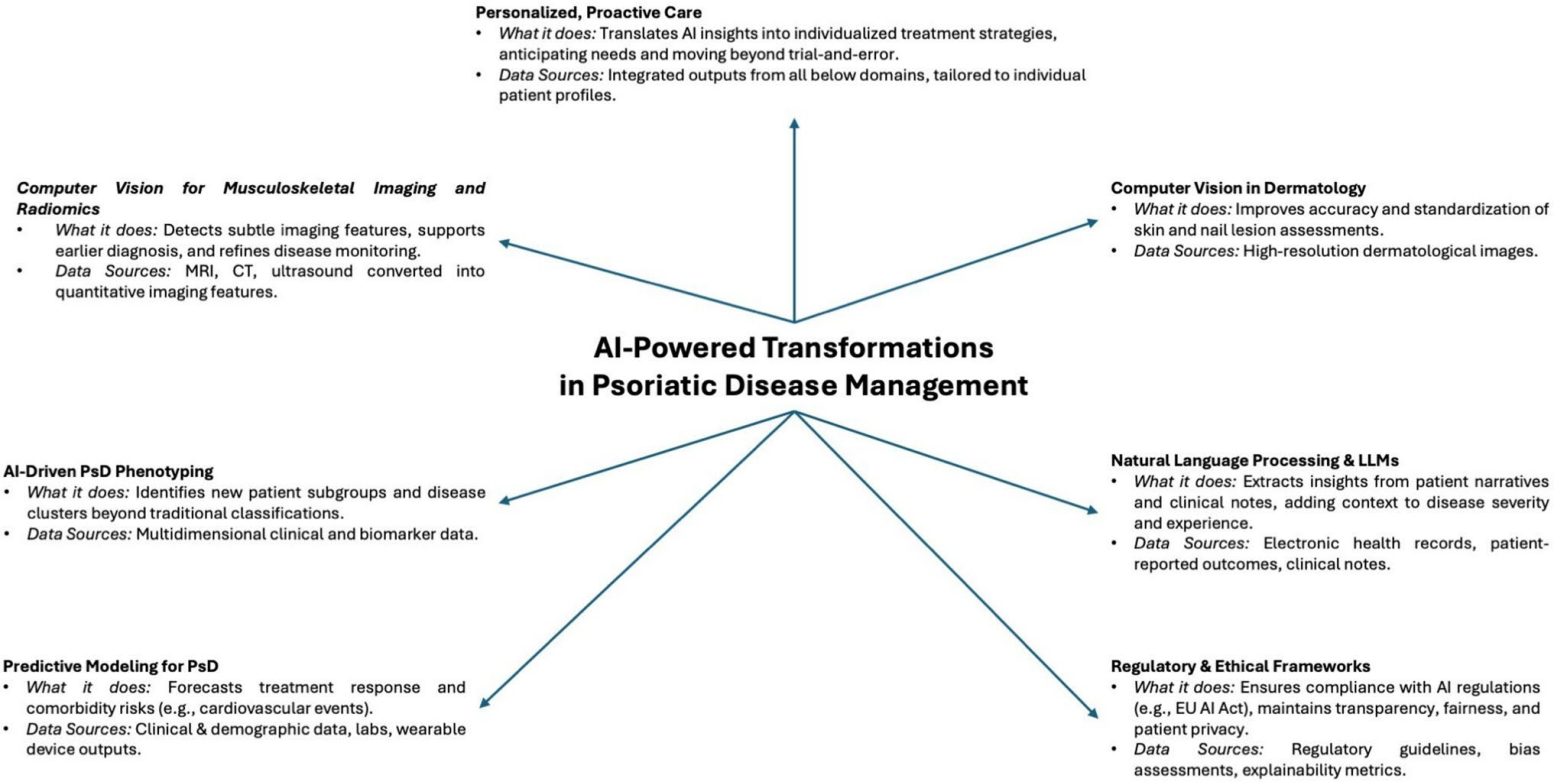
An integrated overview of artificial intelligence-(AI-)driven innovations reshaping psoriatic disease (PsD) management, from predictive modeling and phenotypization to advanced computer vision, radiomics, and natural language processing, all supported by ethical frameworks and culminating in a more personalized, proactive model of patient care

Nevertheless, the true measure of these models’ value lies in their external validation and real-world applicability. Although initial proof-of-concept studies have shown great promise, it remains crucial to demonstrate that these predictive tools can perform reliably across diverse clinical populations, healthcare systems, and practice environments. This necessitates assembling comprehensive, representative datasets—both within and beyond academic centers—and addressing critical issues of data harmonization, standardization, and sharing. Ensuring data quality and uniformity is essential, as subtle differences in how patient attributes are recorded, as well as variations in imaging modalities or laboratory methods, can substantially influence a model’s generalizability.

As predictive modeling continues to evolve, it is likely to benefit from more refined algorithms and the integration of additional data streams, such as genetic predisposition markers and detailed comorbidity profiles. Enhancements in computational infrastructure, including the use of federated learning approaches [7], may enable data scientists to train algorithms across multiple institutions without compromising patient privacy. The end-result could be models that not only predict treatment response or the risk of cardiovascular events but also preemptively identify at-risk individuals, support earlier and more targeted interventions, and ultimately help clinicians move beyond trial-and-error prescribing toward a more precise, proactive approach to PsD care.

The intrinsic heterogeneity of PsD has long challenged our ability to understand its phenotypes and domains, predict outcomes, and determine the most appropriate treatment strategies. Traditional classification systems, heavily reliant on predefined categories or clinician judgment, struggle to capture the nuanced interplay among multiple disease domains, comorbidities, and overlapping pathophysiological processes. In this context, AI-driven phenotypization emerges as a transformative approach capable of revealing hidden disease structures and reshaping how we conceptualize PsD.

Central to this paradigm shift is the application of unsupervised learning methods, which rely on algorithms that detect latent patterns in data without the guidance of established labels or clinical groupings. Unlike conventional approaches that organize patients according to simplified, pre-existing criteria, these techniques can parse immense datasets—including clinical records, imaging features, laboratory results, and even genomic or transcriptomic profiles—and discover meaningful clusters of patients who share previously unrecognized similarities. Through dimension reduction and advanced clustering methods like non-negative matrix factorization, AI can identify distinct phenotypes that better reflect the true complexity of disease biology. For instance, recent analyses of phase III clinical trial data for guselkumab uncovered eight clinically distinct phenotype clusters in bio-naïve PsD patients, each distinguished by unique demographic characteristics and symptom patterns, ranging from joint-dominant phenotypes to those marked by extensive skin involvement or significant enthesitis [8].

The insights derived from such AI-driven phenotypization are more than taxonomic exercises; they have profound clinical implications. By delineating subgroups that differ not only in their primary manifestations but also in their likelihood of responding to certain treatments, clinicians gain the ability to tailor therapeutic strategies to patient-specific needs. It becomes possible, for example, to predict which patients might struggle with conventional systemic therapies due to comorbid obesity or other comorbidities [2, 5], which is known to diminish the efficacy of some biologics, or to identify subgroups at higher risk for cardiovascular complications [6]. Furthermore, integrating comorbidities directly into the clustering process ensures that critical factors such as obesity, cardiovascular risk, depression, or metabolic disturbances are no longer considered peripheral complications but rather integral aspects of the patient’s disease profile [8].

Equally transformative advances are emerging through the integration of computer vision and radiomics, offering prospects that extend beyond what the human eye can discern. Deep learning algorithms trained on MRI, CT, and other imaging modalities have already shown remarkable aptitude for identifying subtle, often imperceptible distinctions in joint structure, bone marrow edema, and soft tissue inflammation that differentiate psoriatic arthritis from other inflammatory arthritides [9–12]. Such refined capabilities could enable earlier detection of disease onset, allowing clinicians to intervene proactively before irreparable joint damage occurs. As these imaging-based models mature, they may also support more meticulous disease monitoring, helping rheumatologists detect subclinical disease activity or subtle changes in disease trajectory and thereby guiding timely modifications to therapeutic regimens. The potential for AI-assisted interpretation of imaging results is not limited to musculoskeletal presentations; in dermatology, computer vision systems such as DeepNAPSI [13] and CAD-PsorNet [14] have demonstrated accuracy and reproducibility rates that rival or surpass the human expert. By standardizing skin and nail assessments—domains that have traditionally been subjective and variable—AI tools can facilitate objective scoring of disease severity, improve inter-rater reliability, and optimize long-term follow-up strategies.

Radiomics, an emerging discipline at the intersection of radiology, data science, and biomedical research, promises to transcend the established boundaries of image interpretation [9, 15]. Instead of relying on purely qualitative assessments, radiomics converts imaging data into a high-dimensional feature space, capturing intricate details of texture, shape, intensity patterns, and other quantitative characteristics invisible to conventional analysis. If validated, such approaches may eventually help characterize and differentiate the imaging signatures of axial PsA from those of other spondyloarthropathies via radiomic analysis on bone marrow edema at sacro-iliac joint site, possibly permitting earlier diagnosis, more precise disease stratification, and more targeted therapeutic interventions. By correlating certain radiomic features with differential treatment responsiveness, this technique could—under the right conditions—offer clinicians invaluable guidance on when to intensify or de-escalate interventions. In principle, this might allow for a shift away from trial-and-error strategies toward more personalized, data-driven management pathways [16, 17].

Another frontier where AI may bring considerable value lies in the realm of natural language processing (NLP) and large language models (LLMs) [18]. While radiomics and computer vision focus on the objective interpretation of images, LLMs excel at deciphering the complexity of human language. Recent explorations have demonstrated that these models can interpret intricate clinical narratives, differentiate nuanced symptom descriptions [19], and, through sophisticated sentiment analysis techniques, illuminate dimensions of patient experience that traditional quantitative measures often overlook. The way patients describe their experiences—subtle linguistic cues in their accounts of pain, daily challenges, or emotional distress—could serve as early indicators of disease progression or evolving therapy response. By mapping these subjective elements onto clinical contexts, clinicians gain a more holistic view of disease impact, potentially intervening earlier or adjusting care pathways to address emerging concerns [20].

Despite their promise, NLP and LLM-driven applications also confront challenges related to accuracy, reliability, and clinical validity. While some studies demonstrate that LLM outputs can approach physician-level comprehensiveness, experienced rheumatologists and dermatologists have identified areas where these models may inadvertently produce misleading or oversimplified interpretations. This underscores the necessity of maintaining a robust human-in-the-loop paradigm, wherein physicians and other healthcare professionals guide model training, validate results, and apply critical judgment to ensure that AI augments rather than replaces clinical reasoning.

The interplay between AI and clinical reasoning deserves particular consideration. On the positive side, AI can enhance critical thinking by providing clinicians with previously inaccessible insights from complex datasets, challenging cognitive biases, and offering alternative interpretations that might not be immediately apparent. By handling routine analytical tasks, AI may free physicians to focus their cognitive resources on more nuanced aspects of patient care that require empathy, ethical judgment, and contextual understanding [21].

However, over-reliance on AI systems could potentially diminish critical thinking skills if clinicians begin to defer too readily to algorithmic recommendations without scrutinizing their foundations. Physicians may find it difficult to critically evaluate conclusions; they cannot fully trace or understand. This tension highlights the importance of designing AI tools that promote transparency and explainability, ensuring that clinicians can meaningfully engage with and critique AI-generated insights rather than simply accepting them at face value.

Such oversight becomes even more critical in the face of stringent regulatory frameworks like the EU AI Act (Regulation (EU) 2024/1689) [22], which classifies clinical decision-support systems as high-risk AI. Meeting these regulatory standards requires transparent model development, bias assessments, traceability of decision-making processes, and ongoing post-market monitoring, ensuring that clinicians remain confident in the safety, accuracy, and fairness of these tools.

The integration of AI into healthcare obliges us to confront critical privacy issues, a matter of particular urgency in large-scale imaging databases and repositories of sensitive patient information. Policies that safeguard patient confidentiality, measures that ensure adequate de-identification, and encryption protocols that protect against breaches must be instituted without hindering innovation. In parallel, frameworks such as the EU AI Act (Regulation (EU) 2024/1689) call for careful classification of AI systems according to their risk profiles, with high-risk applications—those influencing diagnosis or treatment in PsD—subject to rigorous oversight, accuracy standards, and post-market surveillance. Such measures, though stringent, are crucial for fostering trust and ensuring that AI-driven tools adhere to the highest standards of clinical governance.

The path toward clinical implementation faces several significant hurdles that merit careful consideration. Despite promising proof-of-concept studies, many AI models for PsD management require extensive clinical validation in diverse real-world settings, with outcomes rigorously compared against current standards of care. Without this validation across different patient populations, healthcare systems, and clinical contexts, there remains legitimate skepticism about whether impressive laboratory results will translate into actual clinical benefits. The “black box” nature of many deep learning algorithms further complicates clinical adoption, as the lack of interpretability may limit physician trust and regulatory approval, particularly under frameworks like the EU AI Act which emphasizes transparency in high-risk applications.

For PsD management, where genetic information, detailed imaging, and decades of personal health information may be incorporated into AI models, several promising approaches are emerging to address these challenges. The implementation of locally-run algorithms within hospital systems shows particular potential [23, 24]. Edge computing and local LLMs that operate entirely within a healthcare institution’s infrastructure can process sensitive patient data without exposing it to external servers, offering a path to compliance with both regulatory requirements and data protection regulations. These local AI deployments, when combined with federated learning techniques [7], enable model training and improvement across institutions without centralizing sensitive patient data.

Looking ahead, future opportunities for AI in PsD extend beyond current applications. Emerging developments include AI-powered virtual assistants for patient monitoring between clinical visits, integrated multimodal modeling that combines genetic, clinical, and imaging data, and “digital twin” technology that could simulate individual patient responses to different treatment regimens. As the field matures, we may see AI systems capable of identifying pre-clinical markers of disease, potentially enabling interventions that delay or prevent PsD onset in high-risk individuals.

This alignment of clinical innovation with ethical and legal scrutiny paves the way for a future in which clinicians, patients, data scientists, and regulatory authorities collaborate to ensure that AI truly serves the interests of those living with PsD. AI holds the potential to revolutionize PsD management; its success hinges on collaboration: AI to illuminate the unseen and clinicians to guide its application. Together, they can shape a future where complexity not only challenges but empowers care. 

## Authorship statement

Both authors contributed equally in drafting, editing, and revising the manuscript and approved the final version before publication. 
